# *Hydrangea macrophylla* and Thunberginol C Attenuate Stress-Induced Anxiety in Mice

**DOI:** 10.3390/antiox11020234

**Published:** 2022-01-26

**Authors:** Jihye Lee, Huiyoung Kwon, Eunbi Cho, Jieun Jeon, In-Kyu Lee, Wan-Seob Cho, Se Jin Park, Seungheon Lee, Dong Hyun Kim, Ji Wook Jung

**Affiliations:** 1 Division of Endocrinology, School of Medicine, Kyungpook National University, Daegu 41944, Korea; lghlovely@naver.com; 2 Department of Health Sciences, The Graduate School of Dong-A University, Dong-A University, Busan 49315, Korea; kwonhuiyoung@naver.com (H.K.); wcho@dau.ac.kr (W.-S.C.); 3 Department of Pharmacology and Department of Advanced Translational Medicine, School of Medicine, Konkuk University, Seoul 05029, Korea; bee2634@naver.com (E.C.); ji6785@naver.com (J.J.); 4 Department of Internal Medicine, School of Medicine, Kyungpook National University Hospital, Kyungpook National University, Daegu 41944, Korea; leei@knu.ac.kr; 5 Department of Food Biotechnology and Environmental Science, School of Natural Resources and Environmental Sciences, Kangwon National University, Chuncheon 24341, Korea; sejinpark@kangwon.ac.kr; 6 Department of Marine Life Sciences, Jeju National University, Jeju 63243, Korea; slee76@jejunu.ac.kr; 7 Department of Herbal Medicinal Pharmacology, College of Herbal Bio-Industry, Daegu Haany University, Kyungsan 38610, Korea

**Keywords:** *Hydrangea macrophylla*, thunberginol C, corticosterone, restraint stress, anxiety, synaptic plasticity

## Abstract

Stress is an important neurological input for successful life. However, chronic stress and stress hormones could be a cause of various neurological disorders including anxiety disorders. Therefore, there have been many efforts to find effective materials for curing stress-induced neurological disorders. In this study, we examined the effect of *Hydrangea macrophylla* (HM) on corticosterone-induced neurotoxicity, stress-induced anxiety in mice and suggested a possible active ingredient of HM. HM protected cortical neurons against neurotoxicity of corticosterone (CORT), a stress hormone. HM also blocked CORT-induced hippocampal synaptic deficit via regulating Akt signaling. Oral administration of HM improved chronic restraint stress-induced anxiety in Elevated Plus maze test along with reduction of plasma corticosterone and TNF-α levels. Moreover, HM reduced stress-induced neuroinflammation and oxidative stress. Thunberginol C, an active ingredient of HM, also prevented CORT-induced neuronal cell death and restraint stress-induced anxiety. Moreover, thunberginol C reduced plasma TNF-α level and neuroinflammation and oxidative stress. Collectively, HM could be a good candidate for preventing stress-induced neurological disorders and thunberginol C may be an active ingredient of HM for this purpose.

## 1. Introduction

Stress can be either beneficial or harmful to the human body depending on its duration. In the brain, short-term stress is known to enhance cognition, which seems to be related to the long-term potentiation (LTP) improvement effect [1,2,3]. However, long-term stress is known to cause many neuropsychiatric symptoms, such as lowering cognition, increasing anxiety, and even causing depression [4,5,6]. Therefore, the challenge is to develop a variety of neuropsychiatric disorders’ therapeutics through research on materials that could block the changes in brain function caused by long-term stress [7,8,9].

Stress increases the levels of cortisol and norepinephrine in the blood [10,11]. Among them, cortisol is a powerful steroid, and it is known that high concentrations of cortisol can cause neuronal cell death, synaptic function deterioration, and cognitive decline in animals [12,13]. Therefore, there are reports that drugs that block the secretion of cortisol due to stress, or block the cortisol receptor, prevent onset of various neuropsychiatric diseases due to stress, so that drugs targeting them have long been sought [14,15,16].

The leaf of *Hydrangea macrophylla* (LHM) has long been used as a tea having various beneficial effects. Previous studies have reported that LHM has anti-inflammatory and immuno-modulating effects in relation to inflammation and immunity [17,18]. It is also reported that it suppresses pruritus, anaphylactic shock, and allergies by suppressing histamine secretion [19]. In addition, its bladder cancer-cell proliferation inhibition [20], anti-obesity [21], hepatoprotective [22] and anti-diabetic [23,24] effects have been reported. However, studies related to the brain functions have reported only the effect of inhibiting neuroinflammation [17].

Recently, our research team reported that Hydrangeae Dulcis Folium (HM), the fermented and dried leaves of *Hydrangea macrophylla* SER. var. thunbergii MAKINO, suppresses stress-induced cortisol secretion in zebrafish [25]. This suggests that HM could be developed as a new therapy for neuropsychiatric diseases caused by stress. Because zebrafish are genetically different from humans, it is necessary to confirm their effectiveness in animal species that are widely used in preclinical studies in order to develop HM as a pharmaceutical. Therefore, this study tried to verify the effect of HM on stress-induced anxiety in male mice. Previous reports indicated that HM has anti-inflammatory and anti-oxidative effects in mice [17,22]. Moreover, HM suppresses stress-induced cortisol secretion [25]. Therefore, we hypothesized that HM may reduce stress-induced corticosterone secretion, neuroinflammation and oxidative stress. To prove this hypothesis, we observed the effect of HM on serum corticosterone level, microglial activation, and oxidative stress markers in the hippocampus. We first found that HM improved stress-induced anxiety and reduced serum corticosterone level, microglial activation, and oxidative stress. Moreover, thunberginol C, as an active ingredient of HM, showed similar effects with HM on stress. These results suggest that HM and thunberginol C could be candidates for relief of stress-induced psychological disorders.

## 2. Materials and Methods

### 2.1. Materials

Corticosterone and RU486 were purchased from Sigma-Adrich (St. Louis, MO, USA). Corticosterone and TNF-α ELISA kits, and anti-Iba-1 antibody were purchased from Abcam (Cambridge, UK). Thunberginol C was purchased from ChemFaces (Wuhan, China). All other materials were obtained from normal commercial sources and were of the highest grade available.

### 2.2. Animals

CD-1 male mice were purchased from Daehan Biolink (DBL, Eumseong, Korea) and used for experiments after acclimatization to the environment of the experimental animal room at Dong-A University for one week. The environment of the experimental animal room was a temperature of 23 ± 2 °C and a relative humidity of 50 ± 10%, and the light-dark cycle was adjusted in units of 12 h (illumination time 07:00–19:00). Water and feed were ad libitum. Five mice were kept per cage. A total of 120 mice were used in this study (70 for HM study, 50 for thunberginol C study). All animal experiments followed the National Institutes of Health guide for the care and use of laboratory animals (NIH Publications No. 8023, revised 1978) and approved by the Animal Experiment Ethics Committee of Dong-A University (Approval No.: DIACUC-approved-20-6).

### 2.3. HM Preperation

Hydrangeae Dulcis Folium, the fermented and dried leaves of *Hydrangea macrophylla* SER. var. thunbergii MAKINO was purchased from the Han-Nong-Won Co. (Daegu, Korea) and a voucher specimen (JJNUOPS 2014-01) was deposited at the Marine Biomedical Science of the College of Ocean Sciences, Jeju National University. The powder was extracted twice with 70% ethanol at 60 °C for 2 h. The extract (HM) was filtered with a filter paper, then concentrated, and lyophilized (Eyela, model FDU-1200, Japan, yield: 29.18%). We identified the quantity of thunberginol C in the HM was 290 ± 37.5 µg/g [25]. To quantify the thunberginol C, high-performance liquid chromatography (HPLC)-diode array detection analysis was performed using HPLC instrument (Agilent, Waldbronn, Germany), as with the previous report [25].

### 2.4. Primary Cortical Neuronal Culture

The 17-day gestational CD-1 mice were purchased from SAMTAKO (Osan-si, Korea). Animals were anesthetized using isoflurane (2%), and brains were removed from the fetus. Cortical cells were isolated from the fetus, treated with 0.125% trypsin-EDTA (TE), and reacted at 37 °C for 15 min. In order to inactivate TE, it was reacted with Hanks’ balanced salt solution containing 10 mg/L gentamicin and 5% fetal bovine serum (FBS) at room temperature for 10 min. Then, the pellet was rinsed with HBSS without FBS, and the supernatant was removed by centrifugation at 1000× *g* for 5 min. Cells were sufficiently suspended in neurobasal media containing 0.25% glutaMAX, 1% penicillin-streptomycin, and 2% B-27 and cultured at a density of 3 × 10^5^/mL on a poly-L-lysine-coated plate. The medium was half-changed on Day 1 in vitro, and then the medium was exchanged every 3 to 4 days.

### 2.5. Tissue Preparation and Electrophysiology

After anesthetizing the mouse with isoflurane (1.5%), the mouse was sacrificed, and the brain was removed. The brain was submerged into cold artificial cerebrospinal fluid (ACSF; NaCl, 124 mM; KCl, 3 mM; NaHCO_3_, 26 mM; NaH_2_PO_4_, 1.6 mM; CaCl_2_, 2 mM; MgSO_4_, 2 mM; d-glucose, 10 mM), and then the hippocampus was isolated. The hippocampal sections were made with a thickness of 400 μm using a tissue chopper (McIlwain, 121-6), and recovered for 1.5 h–2 h in ACSF maintained at a temperature of 23–26 °C. The hippocampal section was placed in a recording chamber with ACSF at 26–30 °C (flow rate: 2.6 mL/min) and field excitatory postsynaptic potentials (fEPSPs) of the Schaffer collateral-commissural pathway were measured. The fEPSPs response was induced by applying a single electrical stimulation to the Schaffer collateral-commissural pathway. Baseline recordings were recorded for 20 min at a voltage generating amplitude of 40–50% of the maximum amplitude of the fEPSPs. After baseline recording, LTP was induced by high frequency stimulation (HFS; 100 pulses at 100 Hz, 2 times) and recorded for more 1 h. Field potential responses were induced at intervals of 30 s, and the values of slope of fEPSPs were expressed by averaging four consecutive values.

### 2.6. Experimental Schedule

Restraint stress using conical tube (50 mL) was applied to the experimental animals for 4 h a day for 14 d [26]. HM and thunberginol C were suspended in 10% Tween 80 solution and administered daily 1 h before stress application. After 14 days of restraint stress, animal behavior experiment was conducted 24 h after the last stress was applied. One hour after the final test, mice were sacrificed for blood collection and perfusion/fixation of brain.

### 2.7. Elevated Plus Maze

This experiment was conducted with an Elevated Plus-shaped maze consisting of four arms (6 × 36 cm) located at a height of 60 cm [27]. The two opposite arm of the cross maze have walls (closed arm), and the other two have no walls (open arm). When the experimental animal proceeds to an open arm, it will feel anxious because it will see below. Therefore, animals that feel a lot of anxiety spend less time going out onto the open arm. The experimental animals were placed in the center of the maze and the time they spent on each branch was measured using Ethovision (Noldus Information Technology, Wageningen Netherlands) for 5 min.

### 2.8. ELISA

ELISA was performed to measure the concentrations of corticosterone and TNF-α in the blood of animals. The experiment was performed according to the manufacturer’s instructions. First, the animal was anesthetized with isoflurane, an inhalation anesthetic, and blood was collected from the posterior vena cava. To separate the serum, the blood was left at room temperature for 30 min and then centrifuged at 4000× *g* and 4 °C for 15 min. The collected supernatant was diluted 1:20 with assay buffer and used for the experiment. The standards and samples of each factor were mixed with the reaction mixture in a 96-well plate, respectively, and reacted at 37 °C for 30 min in a light-shielding state. By measuring the absorbance at 450 nm using a microplate reader, the concentrations of corticosterone and TNF-α in the blood were compared.

### 2.9. Slice Preparation and Immunohistochemistry

Immediately after blood collection, brains were divided into two hemispheres. One hemisphere was fixed with 4% paraformaldehyde in PB (50 mM, pH 7.4), the other was used for antioxidants’ enzyme measurements. After fixation for overnight, the brain was washed three times with PBS and stored in 30% sucrose solution in PBS (50 mM, pH 7.4). When the brain tissue sank to the bottom, coronal frozen sections were made to a thickness of 30 µm and stored refrigerated in a storage solution (ethylene glycol:glycerol:PB:DW = 3:3:1:3) until the experiment.

Free-floating sections were washed three times with PBS, and reacted with 1% H_2_O_2_ in PBS (50 mM, pH 7.4) for 5 min, then washed three times with PBS and incubated in blocking solution for 2 h (2% normal serum in PBS). Thereafter, they were incubated overnight in the primary antibody solution [rat anti-iba-1 antibody (1:1000 dilution), 0.3% Triton X-100 and 1.5% normal serum. After washing three times with PBS, the sections were incubated in the biotinylated secondary antibody solution (1:1000) for 2 h. After washing three times with PBS, they were then incubated in ABC solution (1:100) for 90 m. After washing three times with PBS, they were then incubated with DAB solution (0.02% 3, 3′-diaminobenzidine and 0.01% H_2_O_2_) for 3 min. The area of cells labeled with Iba-1 antibody was measured with ImageJ program.

### 2.10. Measurement of Lipid Peroxidation

Malondialdehyde (MDA) results from degradation of polyunsaturated lipids. The production of this substance is used as a biomarker to measure the level of lipid peroxidation. MDA reacts with thiobarbituric acid (TBA) as thiobarbituric acid reactive substances (TBARS) to form a 1: 2 MDA-TBA adduct, which is absorbed at 532 nm. Thus, the quantity of TBARS is proportionate to the amount of MDA. The concentration of TBARS was calculated using the MDA standard curve and was expressed as nmol/mg of protein.

### 2.11. Measurements of Antioxidant Enzymes Activity

We used commercially available kits from Randox Laboratories Ltd. (Randox Laboratories, Crumlin, UK) to measure activity of antioxidant enzymes. SOD activity was measured by the method of Goldberg et al. [28] and glutathione peroxidase or glutathione reductase level was measured by the method of Paglia and Valentine [29] as indicated in the kit’s protocol. Enzyme activities were measured with ELISA reader (STAT FAX 3300, Awareness Technologies, MN, USA) and expressed as U/mg protein. 

### 2.12. Statistics

All experiments were evaluated for statistical significance using one-way ANOVA following Tukey post-hoc test. Significance was assessed at the *p* < 0.05 level. Experimental data are expressed as mean ± SD. The raw data are expressed as dots in each graph.

## 3. Results

### 3.1. HM Protected Corticosterone-Induced Neuronal Cell Death and Hippocampal LTP Deficit

**Figure 1:**
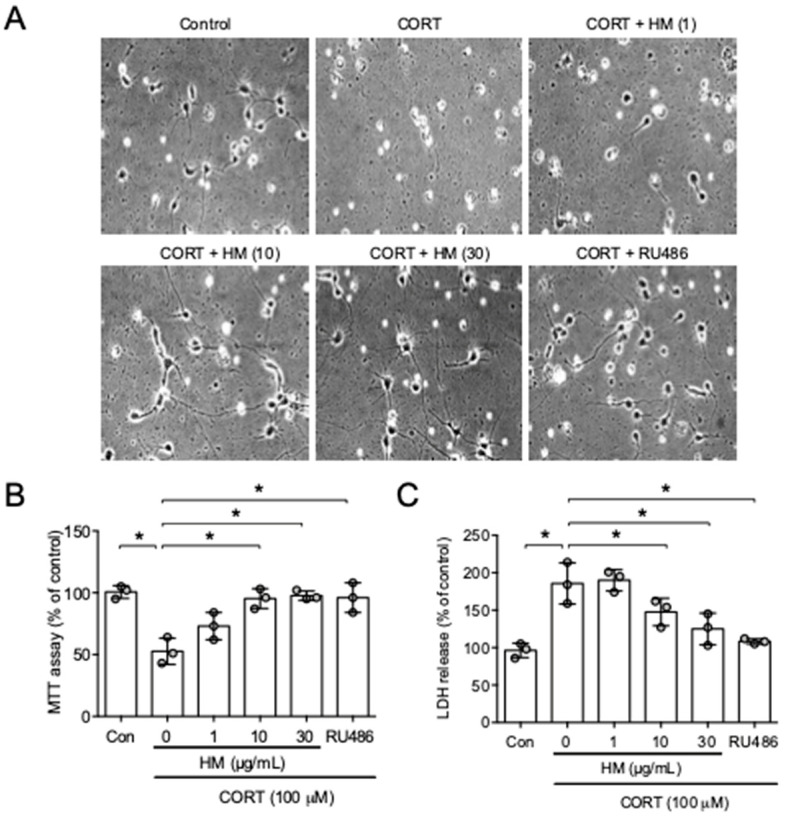
HM protected corticosterone-induced neuronal cell death. Primary cortical neurons were incubated with HM (1, 10, 30 µg/mL) or RU486 (50 µM) for 30 min. Then, the neurons were incubated with corticosterone (CORT, 100 µM) + HM or RU486 for further 24 h. (**A**) Photomicrographs of primary cortical neurons after drug incubation; (**B**) Cells viability observed with MTT assay; (**C**) Cytotoxicity observed with LDH release. Data represented as mean ± SD with raw data. * *p* < 0.05. Con, control. CORT, corticosterone.

To test the effect of HM on stress-induced neurological dysfunctions, we first examined the effect of HM on corticosterone (CORT)-induced neuronal death (Figure 1A). Hippocampal primary neurons were incubated with CORT (100 μM) with HM for 24 h. The CORT-treated group showed significantly lower MTT values and higher LDH levels compared to the control group, suggesting neuronal death. HM (1, 10 or 30 μg/mL) significantly blocked the effect of CORT on neuronal viability (MTT, *F*_5,12_ = 13.57, *p* < 0.05, n = 3/group, Figure 1B; LDH, *F*_5,12_ = 15.13, *p* < 0.05, n = 3/group, Figure 1C). These results suggest that HM protected neuronal cells against CORT challenge.

**Figure 2:**
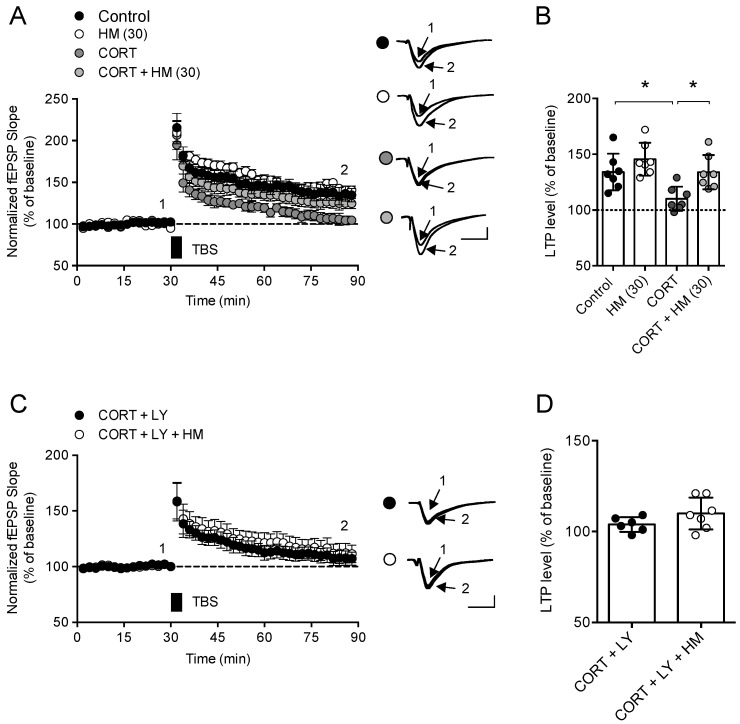
HM prevented CORT-induced LTP deficit through Akt signaling. Hippocampal slices were treated with HM (30 µg/mL) for 30 min, and then CORT (1 µM) + HM (30 µg/mL) for further 2 h before TBS treatment. (**A**) fEPSP level of each group experiment. Data represented as mean ± SEM; (**B**) A bar graph representing the magnitude of LTP (% of baseline) during the last 5 min. Data represented as mean ± SD with raw data; (**C**,**D**) Effect of LY294002 (50 µM) on the effect of HM on CORT-induced LTP deficit. (**C**) fEPSP level of each group experiment. Data represented as mean ± SEM; (**D**) Bar graph representing the magnitude of LTP (% of baseline) during the last 5 min. Data represented as mean ± SD with raw data. * *p* < 0.05. CORT, corticosterone. LY, LY294002.

Next, we tested the effect of HM on CORT-induced hippocampal LTP deficit. In normal hippocampal slices, LTP was induced by HFS (134 ± 8 compared to baseline, n = 7). In the hippocampal slices treated with HM (30 μg/mL) alone, LTP similar to that of normal hippocampal slices was induced (140 ± 5, compared to baseline, n = 7). In hippocampal slices treated with CORT (1 μM) alone, significantly decreased LTP was induced compared to normal hippocampal slices (104 ± 5, compared to baseline, n = 7, *F*_3,24_ = 6.815, *p* < 0.05). In hippocampal slices treated with CORT (1 μM) and HM (30 μg/mL), significantly increased LTP was induced compared to CORT-treated hippocampal slices (104 ± 5, compared to baseline, n = 7, *F*_3,24_ = 6.815, *p* < 0.05, Figure 2A,B). 

Previous reports indicated that corticosterone activated glycogen synthase kinase-3β (GSK-3β) through suppression of phosphoinositide 3-kinases (PI3K)/Akt signaling, in turn suppressed LTP in the hippocampus [30,31]. Therefore, to test if Akt signaling is involved in the effect of HM, we utilized LY294002, a PI3K/Akt signaling inhibitor. LY294002 blocked the effect of HM on corticosterone-induced LTP deficit (*t*_11_ = 1.547, *p* > 0.05, n = 6–7/group, Figure 2C,D).

### 3.2. HM Attenuated Restraint Stress-Induced Increase in Anxiety

**Figure 3:**
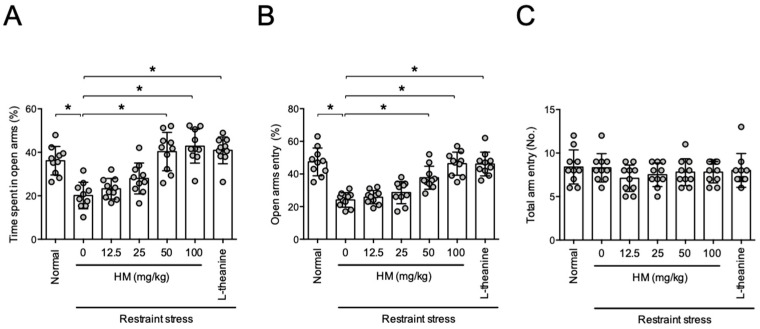
HM prevented stress-induced anxiety. Animals were treated with restraint stress 4 h a day for 14 days. Drugs were administered to the animals 1 h before stress treatment. Elevated maze test was conducted 24 h after the last stress treatment. (**A**) Percentage of time spend in open arms; (**B**) Percentage of open arm entries; (**C**) The number of total arm entry. Data represented as mean ± SD with raw data. * *p* < 0.05.

In the Elevated Plus maze test, restraint stress decreased the percentages of time spent in open arm and open arm entry (time spent, *F*_6,63_ = 7.368, n = 10/group, *p* < 0.05, Figure 3A; entry, *F*_6,63_ = 24.12, n = 10/group, *p* < 0.05, Figure 3B) without affecting the number of total arm entry (*F*_6,63_ = 0.7741, *p* > 0.05, n = 10/group, Figure 3C), suggesting increase in anxiety level. Administration of HM dose-dependently reduced the increase in anxiety in the experimental animals (*p* < 0.05, Figure 3A,B). Administration of 50 and 100 mg/kg of HM showed an anxiolytic effect as similar with L-thiamine, a positive control.

### 3.3. HM Reduced Restraint Stress-Induced Increase of Plasma Corticosterone and TNF-α Level

**Figure 4:**
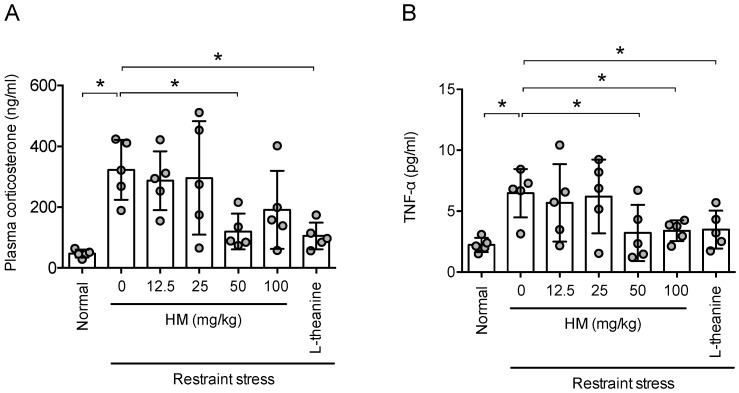
HM prevented stress-induced increase in corticosterone and TNF-α level in plasma. One hour after the Elevated Plus maze test, blood of animals were collected. (**A**) Plasma corticosterone concentration; (**B**) Plasma TNF-α concentration. Data represented as mean ± SD with raw data. * *p* < 0.05.

An increase in blood corticosterone and TNF-α is one of the changes caused by stress in the blood [32]. In a previous study, HM suppressed the rise of cortisol due to stress in zebrafish [25]. Therefore, to determine whether HM suppress the secretion of corticosterone due to stress in rodents, the concentrations of corticosterone and TNF-α in the blood were measured. In this experiment, stress significantly increased the concentrations of corticosterone and TNF-α in the blood, and HM suppressed them in a concentration-dependent manner (corticosterone, *F*_6,28_ = 5.420, *p* < 0.05, n = 5/group, Figure 4A; TNF-α, *F*_6,28_ = 3.124, *p* < 0.05, n = 5/group, Figure 4B). This was similar to the effect of theanine, which is used as a positive control. The above results indicate that HM affects the secretion system of corticosterone.

### 3.4. HM Reduced Restraint Stress-Induced Neuroinflammation

**Figure 5:**
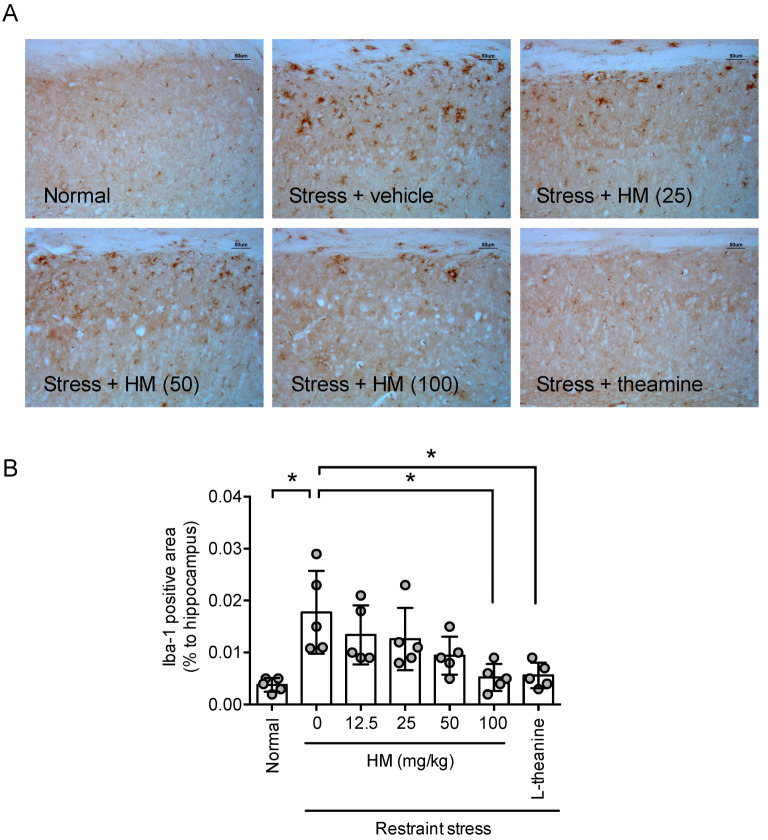
HM prevented stress-induced neuroinflammation. One hour after the Elevated Plus maze test, the animals were sacrificed for immunohistochemistry. (**A**) Photomicrographs of Iba-1-immunopositive cells in the hippocampus; (**B**) Quantitative analysis of Iba-1-positive area in the hippocampus. Data represented as mean ± SD with raw data. * *p* < 0.05.

It has been reported that long-term restraint stress causes cerebral inflammation, leading to a decline in brain function [33]. Therefore, to test whether HM can suppress brain inflammation caused by such stress, changes in microglia cells in the brain were examined. Long-term restraint stress increased the Iba-1-labeled area in the hippocampus (*p* < 0.05, Figure 5A,B). This indicates that the brain inflammation-related cells, microglia, were activated, indicating that neuroinflammation was induced by restraint stress. HM attenuated the increase in Iba-1-labeled area by restraint stress in a dose-dependent manner (*F*_6,28_ = 5.798, *p* < 0.05, n = 5, Figure 5B). Theanine used as a positive control also improved neuroinflammation caused by long-term restraint stress (*p* < 0.05, Figure 5A,B). These results suggest that HM may attenuate stress-induced neuroinflammation.

### 3.5. HM Reduced Restraint Stress-Induced Oxidative Stress

**Figure 6:**
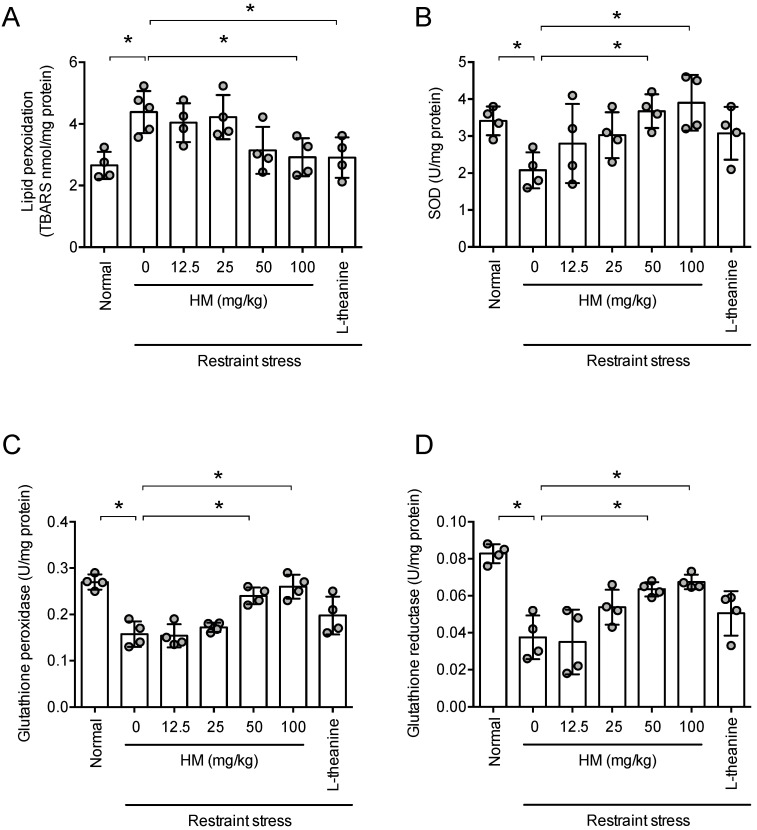
HM prevented stress-induced oxidative stress. One hour after the Elevated Plus maze test, the animals were sacrificed for TBARS and antioxidant enzymes’ activity in the hippocampus. (**A**) TBARS level; (**B**) Superoxide dismutase activity; (**C**) Glutathione peroxidase activity; (**D**) Glutathione reductase activity. Data represented as mean ± SD with raw data. * *p* < 0.05.

Because chronic stress induces oxidative stress in the hippocampus, we tested whether HM can suppress brain oxidative stress caused by such stress. Long-term restraint stress increased TBARS level in the hippocampus (*p* < 0.05, Figure 6A). This indicates oxidative stress was induced by the stress. HM improved this increase in TBARS in a dose-dependent manner (*F*_6,22_ = 5.205, *p* < 0.05, n = 4–5, Figure 6A). Theanine, used as a positive control, also attenuated the increase in TBARS’ levels caused by long-term restraint stress (*p* < 0.05, Figure 6A). Restraint stress significantly decreased activities of superoxide dismutase (SOD, Figure 6B, *p* < 0.05), glutathione peroxidase (Figure 6C, *p* < 0.05) and glutathione reductase (Figure 6D, *p* < 0.05). HM increased the enzyme’s activities in a dose-dependent manner (SOD, *F*_6,21_ = 3.222, *p* < 0.05, n = 4, Figure 6B; glutathione peroxide, *F*_6,21_ = 15.18, *p* < 0.05, n = 4, Figure 6C; glutathione reductase, *F*_6,21_ = 10.90, *p* < 0.05, n = 4, Figure 6D). These results suggest that HM may attenuate stress-induced oxidative stress.

### 3.6. Thunberginol C Protected Corticosterone-Induced Neuronal Cell Death

**Figure 7:**
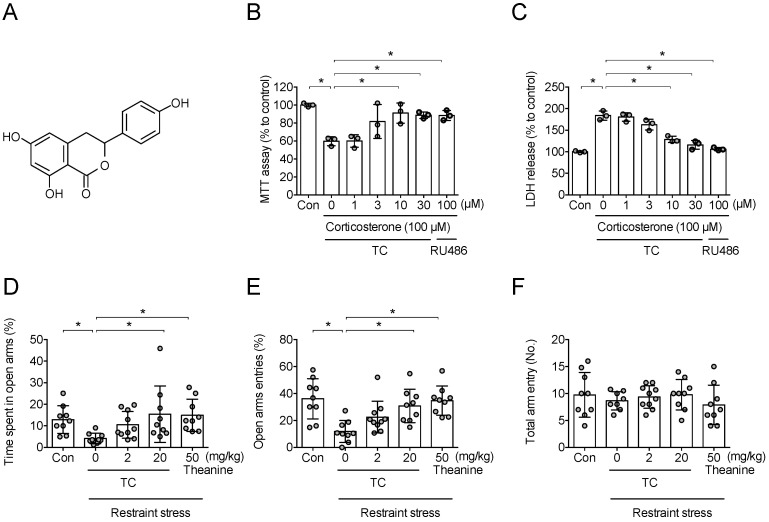
Anti-stress effects of thunberginol C. (**A**) Structure of thunberginol C; (**B**,**C**) Neuroprotective effect of thunberginol C on corticosterone-induced neuronal death; (**B**) Neuronal viability observed with MTT assay; (**C**) Cytotoxicity observed with LDH-release assay. Data represented as mean ± SD with raw data. * *p* < 0.05; (**D**,**E**) Anti-stress effect of thunberginol C on stress-induced anxiety in Elevated Plus maze test; (**D**) Percentage of time spent in open arms; (**E**) Percentage of open arms entry; (**F**) The number of total arms’ entry. Data represented as mean ± SD with raw data. * *p* < 0.05. TC, thunberginol C.

Many previous studies have reported the pharmacological activity of thunberginol C (Figure 7A), isolated from HM. Therefore, to confirm whether the effect of HM shown in this study is the effect of thunberginol C, the anti-stress effect of thunberginol C was examined. As with HM, thunberginol C inhibited corticosterone-induced decrease in MTT (*F*_6,14_ = 8.628, *p* < 0.05, n = 3/group, Figure 7B) and increase in LDH (*F*_6,14_ = 50.05, *p* < 0.05, n = 3/group, Figure 7C) in neuronal cell. This indicates that thunberginol C inhibits corticosterone-induced neuronal death.

### 3.7. Thunberginol C Blocked Restraint Stress-Induced Increase in Anxiety

Next, the anti-stress effect of thunberginol C was examined. In this study, thunberginol C significantly improved the decrease in the percentage of time spent in the open arm (*F*_4,41_ = 3.167, *p* < 0.05, n = 9–10/group, Figure 7D) and open arm entries (*F*_4,41_ = 6.652, *p* < 0.05, n = 9–10/group, Figure 7E) without affecting total arm entries (*F*_4,41_ = 0.2590, *p* > 0.05, 9–10/group, Figure 7F) in the Elevated Plus maze. This indicates that thunberginol C inhibits the increase in anxiety caused by stress.

### 3.8. Effect of Thunberginol C on Plasma Corticosterone and TNF-α Level

**Figure 8:**
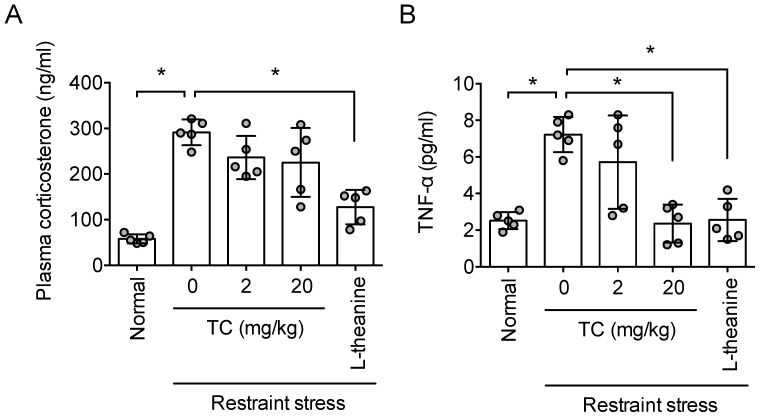
Thunberginol C prevented stress-induced increase in corticosterone and TNF-α level in plasma. One hour after the Elevated Plus maze test, bloods of animals were collected. (**A**) Plasma corticosterone concentration; (**B**) Plasma TNF-α concentration. Data represented as mean ± SD with raw data. * *p* < 0.05. Nor, normal. TC, thunberginol C.

Next, an experiment was conducted to confirm whether thunberginol C could lower the concentration of corticosterone and TNF-α in the blood as with HM. In this experiment, thunberginol C inhibited the increase in blood TNF-α concentration (*F*_4,20_ = 12.56, *p* < 0.05, n = 5/group, Figure 8B) due to stress, but did not inhibit the increase in corticosterone concentration (*F*_4,20_ = 21.33, *p* < 0.05, n = 5/group, Figure 8A). Theanine, used as a positive control, inhibited the increase of corticosterone and TNF-α (Figure 7A,B). This indicates that the inhibitory effect of HM on the increase in blood corticosterone concentration may be due to components other than thunberginol C.

### 3.9. Thunberginol C Suppressed Stress-Induced Neuroinflammation

**Figure 9:**
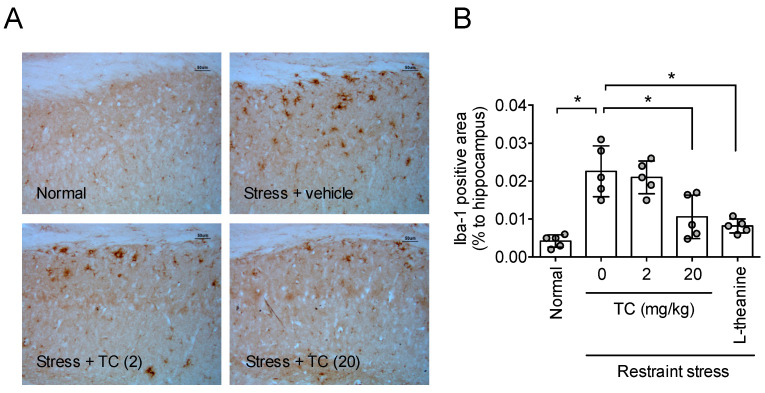
Thunberginol C prevented stress-induced neuroinflammation. One hour after the Elevated Plus maze test, the animals were sacrificed for immunohistochemistry. (**A**) Photomicrographs of Iba-1-immunopositive cells in the hippocampus; (**B**). Quantitative analysis of Iba-1-positive area in the hippocampus. Data represented as mean ± SD with raw data. * *p* < 0.05. TC, thunberginol C.

To confirm whether thunberginol C also improves the neuroinflammation, as with HM, changes in microglia cells in the hippocampus were observed. Long-term restraint stress increased the Iba-1 labeled area in the hippocampus (Figure 9A,B), suggesting that stress caused neuroinflammation. Thunberginol C improved the neuroinflammation in a dose-dependent manner (*F*_4,20_ = 15.94, *p* < 0.05, n = 5/group, Figure 9B). Theanine used as a positive control also improved neuroinflammation caused by long-term restraint stress (Figure 9A,B). This result suggests that thunberginol C attenuates stress-induced neuroinflammation.

### 3.10. Thunberginol C Reduced Restraint Stress-Induced Oxidative Stress

**Figure 10:**
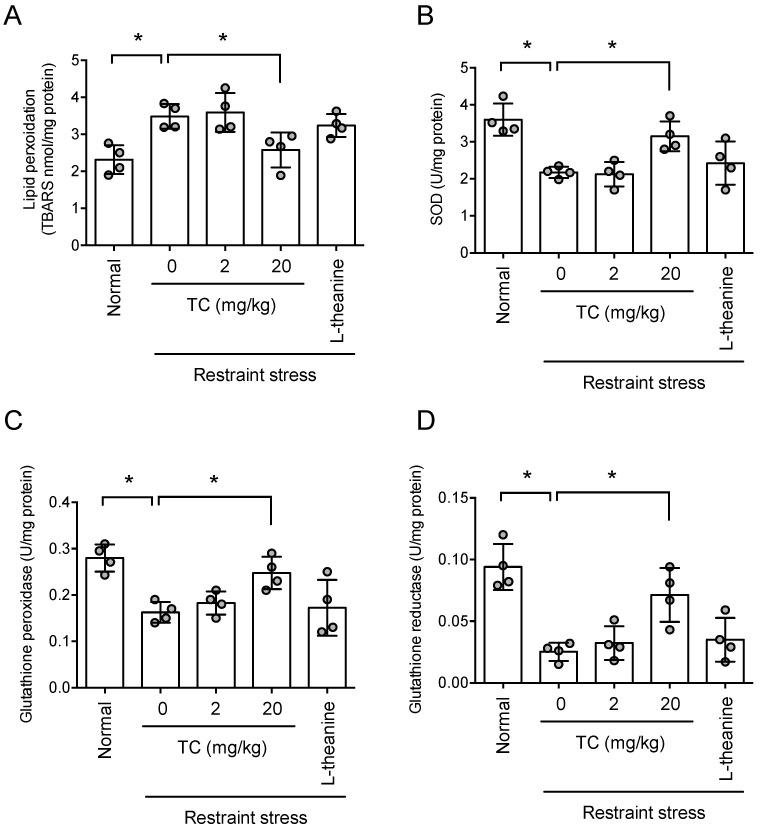
Thunberginol C prevented stress-induced oxidative stress. One hour after the Elevated Plus maze test, the animals were sacrificed for TBARS and antioxidant enzymes’ activity in the hippocampus. (**A**) TBARS level; (**B**) Superoxide dismutase activity; (**C**) Glutathione peroxidase activity; (**D**) Glutathione reductase activity. Data represented as mean ± SD with raw data. * *p* < 0.05.

Because HM reduced oxidative stress, we tested whether thunberginol C also suppressed brain oxidative stress caused by stress. Long-term restraint stress increased TBARS’ levels in the hippocampus (*p* < 0.05, Figure 10A). This indicates that oxidative stress was induced by stress. Thunberginol C improved this increase in TBARS in a dose-dependent manner (*F*_4,15_ = 7.462, *p* < 0.05, n = 4, Figure 10A). Theanine, used as a positive control, also improved the TBARS level caused by long-term restraint stress (*p* < 0.05, Figure 10A). Restraint stress significantly decreased activities of superoxide dismutase (SOD, Figure 10B, *p* < 0.05), glutathione peroxidase (Figure 10C, *p* < 0.05) and glutathione reductase (Figure 10D, *p* < 0.05). HM increased the enzyme’s activities in a dose-dependent manner (SOD, *F*_4,15_ = 10.24, *p* < 0.05, n = 4, Figure 10B; glutathione peroxide, *F*_4,15_ = 7.828, *p* < 0.05, n = 4, Figure 10C; glutathione reductase, *F*_4,15_ = 12.79, *p* < 0.05, n = 4, Figure 10D). These results suggest that thunberginol C attenuates stress-induced oxidative stress.

## 4. Discussion

In this study, HM inhibited corticosterone-induced neuronal death and synaptic plasticity impairment. Since corticosterone is an important substance for inducing various neuropsychiatric diseases due to stress, animal experiments were conducted based on the expectation that HM could improve anxiety symptoms caused by stress. As a result, HM improved the increase in anxiety behaviors caused by stress. HM was also shown to lower the levels of corticosterone and TNF-α in the blood. These effects of HM were almost reproduced by thunberginol C, an active ingredient of HM. 

Corticosterone is an endogenous steroid that acts on both glucocorticoid and mineralocorticoid receptors and is secreted from the adrenal cortex in response to stress [34,35]. Short-term exposure to corticosterone in the central nervous system is known to increase synaptic plasticity [2], improve memory [36,37], and increase neurogenesis [38]. On the other hand, continuous increases in corticosterone or long-term stress inhibit synaptic plasticity [39,40], cause memory loss [41], and inhibit neurogenesis [42], as opposed to short-term exposure. It has also been reported that various neuropsychiatric disorders are caused by prolonged corticosterone elevation [43,44,45]. Several studies have shown that these negative effects are mainly caused by the activity of glucocorticoid receptor (GR) [40,46]. However, the exact mechanism of how GR activation regulates synaptic function is still not clear. Several studies report that signaling mechanisms such as caspase-3, Akt, and GSK-3 are involved in the negative role of corticosterone [30,47]. In this study, it was confirmed that prolonged restraint stress increased blood corticosterone, and it was confirmed that HM and thunberginol C inhibit corticosterone-induced neuronal death. This indicates that HA and thunberginol C have the potential to inhibit negative brain changes caused by long-term stress. 

In a previous study, it was reported that hydrangea extract lowered blood corticosterone levels and reduced the stress response in zebrafish [29]. Corticosterone release is regulated by corticosterone synthesis and up-stream hormones [48,49]. Corticosterone is synthesized from cholesterol through a series of reactions of P450sec, 3β-HSD, 21-hydroxylase, and 11β-hydroxylase [35]. However, we confirmed that HM did not affect the activity of these enzymes (unpublished data). Therefore, it can be hypothesized that HM may be involved in the regulation of corticosterone release. In a previous study, we confirmed that hydrangea extract inhibited ACTH-induced cortisol release in zebrafish [29]. Similarly, in mice, which are higher animals than zebrafish, HM is thought to have an interfering effect on the mechanism related to the release of corticosterone. On the other hand, thunberginol C showed most of the antistress effect of HM, but did not inhibit the increase of corticosterone. This indicates that HM may contain ingredients with anti-stress activity in addition to thunberginol C. HM contains components such as phyllodulcin, hydrangenol, thunberginol A, B, C, D, E, F, and kaempferol and quercetin [50]. Among them, quercetin and kaempferol are known to have antistress and antidepressant effects [51,52]. It has been reported that quercetin can improve various behavioral changes caused by stress and improves depressive symptoms through regulation of BDNF and increase in the amount of serotonin in the brain [51]. Therefore, the antidepressant effect of HM is not due to a single component, but to the complex activity of various components. 

Previous studies have reported that thunberginol C has cholinesterase inhibition [53], soluble epoxide hydrolase inhibition [54], anti-photoaging effects [55], antioxidant and anti-microbial [56,57], and cyclooxygenase inhibition (anti-inflammatory) [55] effects. In this study, it was confirmed that thunberginol C had a protective effect on brain cells and suppressed the expression of anxiety symptoms due to stress. Prolonged stress leads to oxidative damage in the brain and, moreover, to brain inflammation through microglia activation [58,59]. Therefore, previous studies have reported that various substances with antioxidant or brain inflammation inhibitory effects have an improvement effect in stress-induced brain disease models [60,61,62]. Therefore, the brain cell protective and anti-stress effects of thunberginol C confirmed in this study may be related to the antioxidant and anti-inflammatory effects of thunberginol C revealed in previous studies. This can also be supported by the cerebral oxidative stress and inflammation inhibitory effects of thunberginol C confirmed in this study.

In the present study, we found that HM and thunberginol C have anti-stress effects. However they may have different mechanism for those effects. Both previous and the present studies revealed that HM prevented corticosterone elevation by stress [25]. Because corticosterone may cause synaptic deficit, neuroinflammation, and oxidative stress, antistress effects of HM may be due to decrease in corticosterone level [26,27,29]. However, since thunberginol C failed to block the increase in serum corticosterone level by stress, the antistress effect of thunberginol C might be due to its anti-inflammatory and anti-oxidative effects [51,52]. Because HM also prevented corticosterone-induced neuronal death, we cannot rule out the possibility that HM, which contains thunberginol C, has anti-inflammatory and anti-oxidative effects [17,18].

## 5. Conclusions

In this study, we found that HM and its possible active compound, thunberginol C, improved chronic restraint stress-induced anxiety. They also reduced stress-induced increase in serum corticosterone and TNF-α, neuroinflammation and oxidative stress. We still don’t know the bioavailability and BBB penetration rate of thunberginol C, nor do we know whether thunberginol C is the only anti-stress ingredient in HM. However, HM and thunberginol C are effective in stress-induced anxiety, and prolonged administration of them did not show any detrimental effects on mice physiology including weight and total arm entries in the Elevated Plus maze test. Therefore, we suggest them as a candidate for functional food and medicine for stress-induced psychological disorders.

## Data Availability

Data are contained within the article.
